# Evolutionary relationships between metabolism and behaviour require genetic correlations

**DOI:** 10.1098/rstb.2022.0481

**Published:** 2024-02-26

**Authors:** Amélie Crespel, Jan Lindström, Kathryn R. Elmer, Shaun S. Killen

**Affiliations:** ^1^ Department of Biology, University of Turku, Turku 20014, Finland; ^2^ School of Biodiversity, One Health, and Veterinary Medicine, College of Medical, Veterinary and Life Sciences, University of Glasgow, Glasgow G12 8QQ, UK

**Keywords:** evolution, indirect selection, metabolic rate, physiology, genetic (co)variance

## Abstract

As selection acts on multivariate phenotypes, the evolution of traits within populations not only depends on the genetic basis of each trait, but also on the genetic relationships among traits. As metabolic rate is often related to vital traits such as growth, physiology and behaviour, its variation and evolution is expected to have important repercussions on individual fitness. However, the majority of the correlations between metabolic rate and other traits has been based on phenotypic correlations, while genetic correlations, basis for indirect selection and evolution, have been overlooked. Using a case study, we explore the importance of properly estimating genetic correlations to understand and predict evolution of multivariate phenotypes. We show that selection on metabolic traits could result in indirect selection mainly on growth-related traits, owing to strong genetic correlations, but not on swimming or risk-taking and sociability behaviour even if they covary phenotypically. While phenotypic correlation can inform about genetic correlation direction, caution is needed in predicting the magnitude of genetic correlation. Therefore, even though phenotypic correlations among physiological and behavioural traits could be useful, deriving evolutionary conclusions based purely on them is not robust. In short, proper estimation of genetic correlations is needed when predicting evolutionary consequences.

This article is part of the theme issue ‘The evolutionary significance of variation in metabolic rates’.

## 1. Introduction

For evolution to occur, specific phenotypes must be under selection and their genetic basis transmitted to the next generation [1]. As individuals are the result of interactions among a large variety of traits, the phenotypic response to selection will depend not only on the genetic basis of independent traits but also on the genetic relationship among the traits [2–5]. A selection pressure on one trait can then indirectly lead to selection and phenotypic change in a correlated trait, even if the correlated trait itself is not under direct selection [2]. As selection acts on multivariate phenotypes, it is necessary to document indirect selection pressures and correlation structures that different traits may experience, to better understand and predict the evolutionary potential of the phenotypes in a population.

Metabolic rate can be viewed as a quantification of the energetic cost of living and the rate at which energy can be used to support biological functions [6]. Therefore, metabolic rate is an integrative trait that is often related to other key traits potentially altering the fitness of individuals, including growth, reproduction, digestion, movement and behaviour [6]. The pace-of-life syndrome (POLS) hypothesis describes covariation among aspects of physiology, life history and behaviour, and therefore predicts that trait associations will define correlated evolutionary responses [7]. In line with these predictions, individuals with higher metabolism tend to grow faster when there is sufficient food, reproduce earlier, take more risks and have shorter lifespans [8–11]. This is because a high metabolic rate needed to support faster growth would also incur higher maintenance costs, with a need for high energy intake and thus riskier behaviour to access resources, leading to increased mortality risks [12]. Inter-individual variation and evolution of metabolism is thus expected to have important repercussions on a variety of associated physiological and behavioural traits, ultimately affecting the survival and ecology of populations. Indeed, over the last decade or so, there has been a surge of research interest in how metabolic phenotypes relate to behaviour, life-histories and fitness, with a particular emphasis on understanding the role of energy use in coping with environmental change [6,11,13,14]. Notably, however, the majority of the covariations previously estimated have been based on phenotypic correlations, with relatively little known about the genetic correlation among the traits being considered (but see [15,16]). Therefore, most speculation regarding the evolutionary implications of links between metabolic rate and other traits has been conducted without knowing if the observed phenotypic correlation actually corresponds with the underlying genetic correlation.

Information on heritability and genetic correlations is necessary to properly understand the extent to which indirect selection can have evolutionary consequences. While this requirement has been understood for decades [2,17,18], it is frequently overlooked in eco-physiological studies of metabolic rates. Genetic correlation can arise owing to pleiotropy or linkage disequilibrium and reflects how much of the genetic variance of one trait has a causal effect or is associated with the expression of another [2,4,5,17,19]. In pleiotropy, a single gene is involved in the regulation and genetic pathway of multiple different traits [4]. Linkage disequilibrium happens when two genes affecting two different traits tend to be inherited together, either by chance if the two genes are physically close in the genome [20] or when past selective pressures favoured a particular combination of phenotypic traits [5]. Genetic correlations can thus prevent traits from evolving independently [21] and, without genetic correlation, covariation among traits is context-dependent and not transmitted to the next generation [2,17]. The genetic correlation structure can thus constrain or facilitate the response to selection [3,22–24], ultimately affecting the evolutionary trajectory of populations across the adaptive landscape. Indeed, genetically correlated traits can reduce the pace at which beneficial traits are fixed if the traits are under different selection pressures, but accelerate fixation if both traits are under the same selection pressure. Genetic correlations are thus the basis for indirect responses to selection and evolution but those between metabolism and other fitness-related traits are in general poorly documented. This lack of knowledge probably originates from the difficulty in estimating genetic correlations, because it requires large sample sizes and information about the relatedness among individuals [17].

As genetic correlations are difficult to estimate, there is often the assumption that phenotypic correlations may be a good substitute. This assumption is referred to as the phenotypic gambit in the behavioural ecology literature [25], or Cheverud's conjecture more broadly [26]. Phenotypic correlations have been considered good substitutes for genetic correlations particularly among morphological traits, between morphological and life-history traits, and among behavioural traits, but rarely among life-history traits [22,23,27–29]. However, despite generally strong associations between phenotypic and genetic correlations, the absolute difference between the two correlations can also be high, with a lack of precision in the genetic correlation estimates derived from the phenotypic correlations [27,29]. Because phenotypic correlations can be decomposed into genetic and environmental components [2,17], changes in phenotypic correlations might not always reflect changes in genetic correlation. This would be the case particularly if environmental effects can mask low or negative genetic correlations between traits because of trade-offs among the traits [15,30]. For example, regarding the POLS, Santostefano *et al.* [15] revealed that the syndrome was not supported by genetic correlations, as ‘risky’ behaviours did not mediate life-history trade-offs between developmental time and lifespan, with environmental effects masking the genetic constraints between developmental time and lifespan. Therefore, it is still unclear how variation in metabolism could affect the physiological and behavioural traits of a population across generations, as their genetic relationship has not yet been rigorously examined.

Here, we explore the importance of properly estimating genetic correlations to understand and predict evolution of multivariate phenotypes. Specifically, we document the phenotypic and genetic correlations among traits associated with metabolism, and putatively related physiological and behavioural traits, and discuss the potential consequences of evolution in metabolic rate on the other fitness-related traits. We also describe the relationship between the phenotypic and genetic correlations, and examine the significance of using the phenotypic gambit with metabolic traits in cases where genetic correlations are difficult to assess. The approach we use is not new, as there are classical examples examining the role of genetic correlations in indirect selection using tools in quantitative genetics [2,18]. To date, however, there is a lack of worked examples comparing genetic and phenotypic correlation using metabolic traits, and an over-reliance on assuming that phenotypic correlations are an adequate substitute for genetic correlations in the study of metabolic phenotypes, without any explicit testing of this assumption. Despite the paucity of information in this area, metabolic traits may be ideal for illustrating the importance of differences in genetic and phenotypic correlations for selection and potential evolutionary change. First, while correlations between metabolic traits and aspects of behaviour and life-histories are known to occur, these correlations are highly labile which suggests a strong environmental contribution to them which are often underappreciated within the context of specific studies examining phenotypic correlations. Second, the heritability of metabolic traits can often be low, and itself dependent on environmental context, suggesting that correlations between metabolic rates and other traits may not be passed on to the next generation despite the presence of phenotypic correlations. Finally, the strong correlation between metabolic rates and body mass can lead to inflated estimates of phenotypic correlations if not properly accounted for, potentially causing overestimations of the assumed genetic links between traits and potential responses to selection.

As a case study, we use metabolism, physiological and behavioural phenotypic data from a population of zebrafish (*Danio rerio*), that allowed the large sample size needed for precise estimation of genetic correlations [17]. Although some previous studies have examined additive genetic variance in metabolic rates [16,31], none have also examined genetic and phenotypic correlations across a large range of behaviours and measures of performance (e.g. locomotor capacity and growth). Our intention is to highlight that the use of only phenotypic covariance estimates among physiological and behavioural traits limits the robustness of evolutionary conclusions. This work can act as a roadmap for the many researchers investigating links between metabolic rates and behavioural traits, illustrating how to compare phenotypic and genetic correlations, and illustrating the important differences that can occur.

## 2. Metabolic traits phenotypically covary with physiological and behavioural traits

To explore the link between metabolic traits and physiological or behavioural traits, we used 24 adult zebrafish from a semi-wild population (about five generations in captivity before arriving at the University of Glasgow, sourced from rearing ponds in Malaysia, with expected heterozygosity in the range of natural populations, *H*_e_ = 0.25) to create 36 families in a controlled factorial (North Carolina II) design, where four groups of three males were reciprocally crossed with three females (i.e. nine families per group). After rearing to adulthood, we screened about 800 fish (20–25 fish per family) for metabolic traits (standard metabolic rate (SMR) and maximum metabolic rate (MMR)), as well as physiological traits (growth, and critical swimming speed (*U*_crit_)) and behavioural traits (risk-taking behaviour, measured as speed when moving in an open field, average distance from the centre of an open field, distance moved in an open field, number of emergences from a shelter during a 10 min trial and sociability, measured as average distance from a stimulus shoal of conspecifics) (see [32,33] for previous results and details on the methodology).

### (a) Positive phenotypic covariation between metabolic traits and physiology

**Figure 1.**
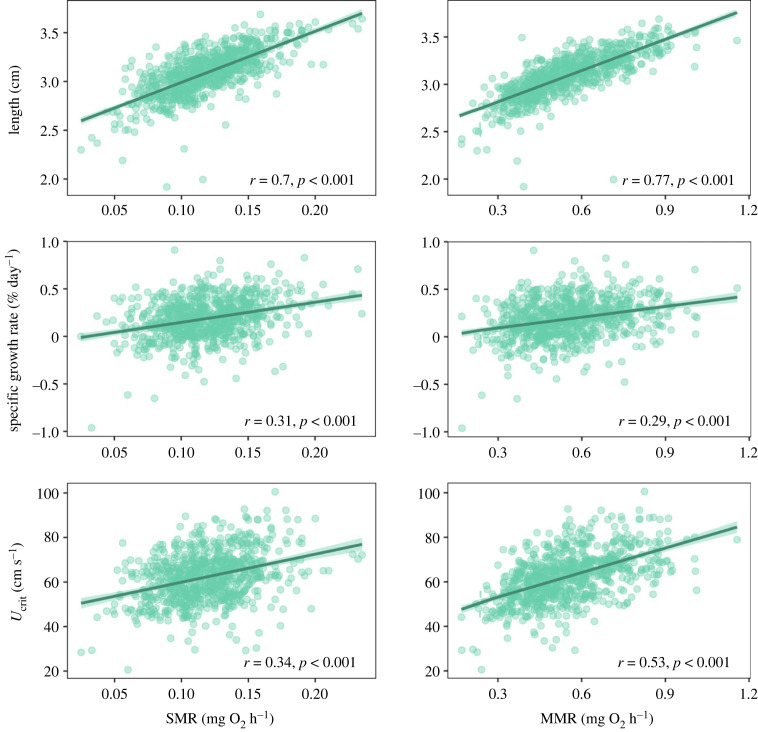
Phenotypic correlation between standard metabolic rate (SMR, on the left) or maximum metabolic rate (MMR, on the right) with the physiological traits total length, specific growth rate and critical swimming speed (*U*_crit_), *n* = 797. For illustration purposes, we also presented the lines of best fit between the traits (the shaded areas around the lines correspond to 95% confidence intervals); we do not imply causal relationships by them.

Phenotypic correlations were indeed observed between the metabolic and physiological traits. Using SMR and MMR without adjusting to the average body mass of the fish (as we might expect potential selection to operate on the entire integrated phenotype), we showed a strong positive phenotypic correlation between SMR and MMR (*r* = 0.68, *p* < 0.001). SMR also had a strong positive phenotypic correlation with total length (*r* = 0.70, *p* < 0.001), and moderate positive phenotypic correlation with growth and *U*_crit_ (*r* = 0.31 and *r* = 0.34, *p* < 0.001, respectively) (figure 1). Similarly, MMR had strong positive phenotypic correlation with length (*r* = 0.77, *p* < 0.001), and moderate positive phenotypic correlation with growth and *U*_crit_ (*r* = 0.29, and *r* = 0.53, *p* < 0.001, respectively). Fish with higher SMR or MMR were also longer fish with higher growth rate and swimming speed (figure 1).

When SMR and MMR were adjusted to the average body mass of the fish (0.30 g), the two traits still had a moderate positive phenotypic correlation to each other (*r* = 0.23, *p* < 0.001). While mass-adjusted SMR was no longer correlated with total length (*r* = 0.06, *p* = 0.073), there was still a significant positive phenotypic correlation between SMR and each of growth and *U*_crit_ (adjusted for length), although both were weak (*r* = 0.10, and *r* = 0.09, *p* = 0.004 and *p* = 0.012, respectively) (electronic supplementary material, figure S1). Mass-adjusted MMR was still significantly positively correlated with length, growth and length-adjusted *U*_crit_, but the phenotypic correlation was weak with length and growth (*r* = 0.22, and *r* = 0.11, *p* < 0.001 and *p* = 0.002, respectively), while still moderate with *U*_crit_ (*r* = 0.35, *p* < 0.001) (electronic supplementary material, figure S1). Body mass, therefore, seemed to be an important driver of phenotypic correlations between metabolic traits and growth, but not with *U*_crit_.

Overall, the phenotypic correlations we observed were in line with the predictions of the POLS and common expectations that higher metabolic rates would support aerobically demanding functions such as growth or swimming performance [14,34,35]. However, a lack of phenotypic correlation between growth and SMR has also been reported [11,36,37]. Here, we showed that, in our population, not only was MMR positively correlated with the energetically demanding non-maintenance functions we measured, but also that SMR was correlated with these functions. A high SMR reflects the maintenance costs of a greater metabolic machinery able to accommodate the additional physiological tasks [6,10,38]. Both growth and swimming can affect condition and survival of the fish, especially regarding foraging needs and capacity to access new resources, and predator susceptibility and avoidance [39]. The phenotypic correlation between these traits and the metabolic traits could then accelerate their possible response to selection especially if the traits possess genetic (co)variation.

### (b) Positive but weak phenotypic covariation between metabolic traits and behaviour

**Figure 2.**
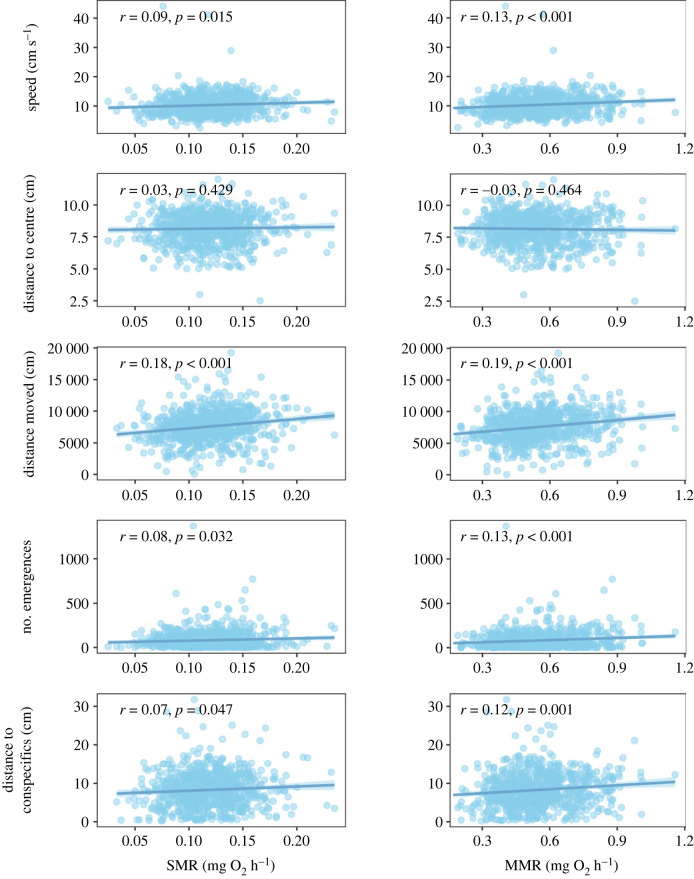
Phenotypic correlation between standard metabolic rate (SMR, on the left) or maximum metabolic rate (MMR, on the right) with the behavioural traits reflecting risk-taking (speed when moving in an open field, distance from centre of an open field, distance moved in an open field, number of emergences from a shelter) and sociability (distance to conspecifics), *n* = 797. As in figure 1, the lines of best fit between the traits are presented for illustration purposes only.

Phenotypic correlations were also generally observed between metabolic and behavioural traits. SMR had weak but significant positive phenotypic correlation with total distance moved (*r* = 0.18, *p* < 0.001), spontaneous movement speed (*r* = 0.09, *p* = 0.015), shelter emergences (*r* = 0.08, *p* = 0.032) and sociability (*r* = 0.07, *p* = 0.047), and no phenotypic correlation with distance from centre of the open field (*p* = 0.43) (figure 2). Similarly, MMR had a weak but significant positive phenotypic correlation with total distance moved, speed, shelter emergences and sociability (*r* = 0.19, *r* = 0.13, *r* = 0.13, and *r* = 0.12, *p* < 0.001, respectively), and no phenotypic correlation with distance from centre (*p* = 0.46). Fish with higher SMR or MMR were fish showing higher risk-taking and lower sociability behaviour (figure 2).

When SMR and MMR were mass-adjusted, the strength of phenotypic correlations with behaviour decreased. Mass-adjusted SMR was then significantly but only weakly correlated with sociability (*r* = 0.08, *p* = 0.032) (electronic supplementary material, figure S2). Mass-adjusted MMR was no longer correlated to total distance moved (*p* = 0.125) but had still significant weak phenotypic correlation with speed, shelter emergences and sociability (*r* = 0.08, *r* = 0.10 and *r* = 0.14, *p* = 0.017, *p* = 0.005 and *p* < 0.001, respectively) (electronic supplementary material, figure S2). Body mass seemed thus to be a main driver of the phenotypic correlations between metabolic traits and risk-taking behaviour, but not sociability.

These results were again mainly in line with the predictions of the POLS, even if the phenotypic correlations observed here were weak or mass-dependent, especially for the correlation between SMR and risk-taking. Previous studies in fish found a significant phenotypic relationship between metabolic rates, especially SMR, and risk-taking behaviour, measured as boldness or activity level [11,12,40,41]. However, many other studies also reported limited support for the POLS, with unclear phenotypic correlations between metabolic traits and risk-taking behaviour [12,41,42]. Consistent with our results, some authors have found that body length—more than metabolic rate—influenced risk-taking behaviour [43]. Our results are consistent with the prediction that higher metabolic rate, both SMR and MMR, would lead to less social fish, because of the competition for resource acquisition to support the metabolic demand [6,44]. While this phenotypic relationship has previously been observed for SMR [45,46], see [44] for a negative correlation between sociability and MMR. Overall, our results support the idea that SMR and MMR could indeed be positively related to behaviour that would require a higher level of energy expenditure, following the ‘performance model’, whereby increased metabolic machinery and associated high maintenance costs have high capacity for energetically demanding behaviour [9,10]. As both risk-taking and sociability behaviour are linked with foraging trade-offs between access for food or protection, they may have an impact on survival and fitness of individuals, with the phenotypic correlations (even if weak) accelerating possible responses to selection if leading to synergetic effect on the selection of the traits.

## 3. Limited genetic correlation between metabolism and physiology or behaviour

To estimate the genetic correlations among the different traits and, therefore, advancing the understanding of their evolutionary dynamics, we used a quantitative genetics statistical approach [2,17]. Genetic variances and covariances between SMR or MMR and the physiological or behavioural traits were determined using the animal model [17,19], accounting for common environment and parental effects, as well as sex, using the software ASReml [47]. The total phenotypic variance (*V*_P_) of each trait was decomposed into additive variance (*V*_A_), common environment and parental variance (*V*_CEP_) and residual variance (*V*_R_) allowing the estimation of the narrow-sense heritability (*h*^2^) as the ratio of the additive variance to the total phenotypic variance (*h*^2^ = *V*_A_/*V*_P_). Genetic (*r*_G_) correlations were estimated using bivariate models including two traits as response variables. A likelihood ratio test was used to evaluate the significance of the genetic components (additive genetic (co)variance) estimated by comparing the full model with a model in which the (co)variance was set to zero.

### (a) Genetic (co)variance structure of metabolism, physiology and behaviour

Even though metabolic rates were phenotypically correlated to most physiological and behavioural traits (figures 1 and 2), very few genetic correlations were observed. Even so, the heritability estimates for all traits were significantly greater than zero, except for SMR (table 1). However, the heritability of MMR, length, *U*_crit_, speed when moving, distance from centre, and shelter emergences were low (ranging from 0.08 to 0.11), while the heritability of growth, distance moved and sociability were moderate (0.16, 0.22 and 0.23 respectively). The heritability estimates of the physiological traits were low compared to those previously reported in other fish species (ranging from 0.20 to 0.60) [48,49] but the heritability of the behavioural traits were in the same range (0.10–0.45) [50,51]. Generally, behavioural traits have a moderate heritability, lower than 0.3 [27]. Here, we found that the behavioural traits did not have lower heritability than the metabolic or physiological traits. Therefore, depending on the selection pressure applied, all these traits, except SMR, could potentially evolve across generations. However, as their heritability is low or moderate, the rate of the evolutionary response would be low and the phenotypic expression of the traits, and the links among them, could be strongly influenced by environmental effects.

**Table 1.** Heritability (*h*^2^ (s.e.)) and genetic correlations (Gen corr (s.e.)) with 95% confidence intervals (CI) of the metabolic (SMR and MMR), the physiological (total length, growth, swimming) and the behavioural (speed when moving, distance from centre, distance moved, number of emergences, distance to conspecifics) traits. (Dark blue cells indicate significant heritability or genetic correlations (*p* < 0.05), light blue cells indicate marginal trend for positive correlation and yellow cells for negative genetic correlation (*p* = 0.05–0.10).)

	*h*^2^	*p-*values	GenCorr SMR	95% CI (lower – upper)	*p-*values	GenCorr MMR	95% CI (lower – upper)	*p-*values
standard metabolic rate, SMR	0.04 (0.04)	0.286				0.92 (0.38)	(0.17 – 1.67)	0.095
maximum metabolic rate, MMR	0.08 (0.05)	0.021	0.92 (0.38)	(0.17 – 1.67)	0.095			
length	0.09 (0.06)	0.036	0.93 (0.33)	(0.29 – 1.57)	0.072	0.96 (0.09)	(0.78 – 1.14)	0.029
specific growth rate	0.16 (0.08)	0.002	0.65 (0.41)	(−0.15 – 1.45)	0.209	0.71 (0.28)	(0.17 – 1.25)	0.056
swimming, *U*_crit_	0.09 (0.05)	<0.001	0.13 (0.50)	(−0.84 – 1.10)	0.788	0.47 (0.30)	(−0.12 – 1.06)	0.209
speed	0.11 (0.06)	0.003	0.09 (0.51)	(−0.91 – 1.09)	0.854	−0.09 (0.39)	(−0.87 – 0.69)	0.816
distance from centre	0.08 (0.04)	0.002	−0.99 (0.51)	(−1.98 – 0.00)	0.054	−0.55 (0.41)	(−1.34 – 0.24)	0.140
distance moved	0.22 (0.08)	<0.001	0.45 (0.39)	(−0.32 – 1.22)	0.671	0.30 (0.32)	(−0.33 – 0.93)	0.377
number of emergences	0.10 (0.05)	0.007	0.17 (0.52)	(−0.85 – 1.19)	0.752	0.19 (0.40)	(−0.60 – 0.99)	0.655
distance to conspecifics	0.23 (0.09)	<0.001	0.43 (0.45)	(−0.45 – 1.31)	0.697	0.41 (0.32)	(−0.21 – 1.03)	0.232

Most phenotypic correlations previously highlighted were not supported at the additive genetic level. Only the genetic correlation between MMR and total length was significant, positive and strong (*r*_G_ of 0.96; table 1). Thus, individuals with a genetic basis for higher MMR outcome also had a genetic basis for being longer. Still, some other genetic correlations were marginally significant and potentially biologically relevant. There was a tendency of genetic correlations between SMR and length, SMR and MMR and MMR and growth (*p* = 0.05–0.10), highlighting potentially strong positive genetic correlation (*r*_G_ of 0.93, 0.92 and 0.71, respectively), while SMR and distance from centre displayed marginal strong negative genetic correlation (*r*_G_ of −0.99). For those physiological traits, individuals genetically predisposed for higher metabolic rates would also have the tendency to be predisposed for longer size or growth. On the other hand, individuals predisposed for higher maintenance cost (SMR) would also have the tendency to be predisposed for less exploratory or risk-prone behaviour.

### (b) Genetic structure and prior evolution

The genetic correlation among traits can provide insight into the genetic structure of the traits but also past evolutionary processes. Here, total length and growth displayed genetic correlations with metabolic rates, possibly arising owing to pleiotropy or linkage disequilibrium [2,17,19]. While genetic correlation arising from linkage disequilibrium can be altered because of recombination or changes in selective pressures, genetic correlations arising from pleiotropy will persist across generations [24]. Here, it is difficult to speculate which past evolutionary processes led to the genetic correlation among the traits we observed, and investigation of the genetic correlations across generations is needed. However, the absence of genetic correlations between the metabolic rates and swimming performance or behaviour suggests that those traits were supported by different sets of genes. Importantly, analysing phenotypic correlations on their own would not allow distinguishing between the alternatives of different sets of genes affecting the same trait, owing to either pleiotropy or linkage disequilibrium.

### (c) Limited potential for future correlated evolution

Genetic correlations among traits can also inform the potential for future correlated evolution, with responses to indirect selection and evolutionary constraints [11,22]. Indirect evolution could thus occur when considering MMR and total length, with direct selection for higher MMR expected to lead to indirect selection for longer fish, even in the absence of direct selection on length itself. In a similar way, even if only marginally significant, selection favouring higher MMR would favour faster growing fish, and selection favouring higher SMR would favour longer and more explorative fish. As length and growth are traits particularly affecting fish survival, there would be a strong potential for accelerating metabolic rate evolution. However, the presence of genetic correlations may also slow adaptive responses of traits by preventing the traits from becoming independently optimized by selection, if different selection pressures affect each trait, resulting in potential trade-offs among traits [3,11,22,29]. Increased knowledge of the selection regime on metabolic rates, length and growth would be needed to better predict the evolutionary consequences.

Here, it is clear that selection on either SMR or MMR will be unlikely to induce indirect selection or correlated evolutionary responses in *U*_crit_ or behaviour owing to the lack of significant genetic correlations. MMR may thus not solely evolve owing to pressure on swimming performance or exercise, but perhaps to other traits related to the maximization of aerobic scope [52], though this perspective has rarely been considered in studies of aerobic capacity. Therefore, the influence of metabolism on the evolutionary trajectory of swimming and behaviour is lower than what was suggested by the phenotypic correlations and thus more likely the result of independent direct selection on each trait rather than indirect selection response from evolution of the metabolic traits [11,31]. This lack of indirect selection could be an advantage for the fish as all the traits could then be independently optimized by selection, without constraints and the need to favour one trait over the other. For example, depending on the selection pressure, traits could respond in opposite directions, favouring the best phenotypic outcome for the specific environment.

### (d) Significance of phenotypic correlations that are not supported by genetic correlations

**Figure 3.**
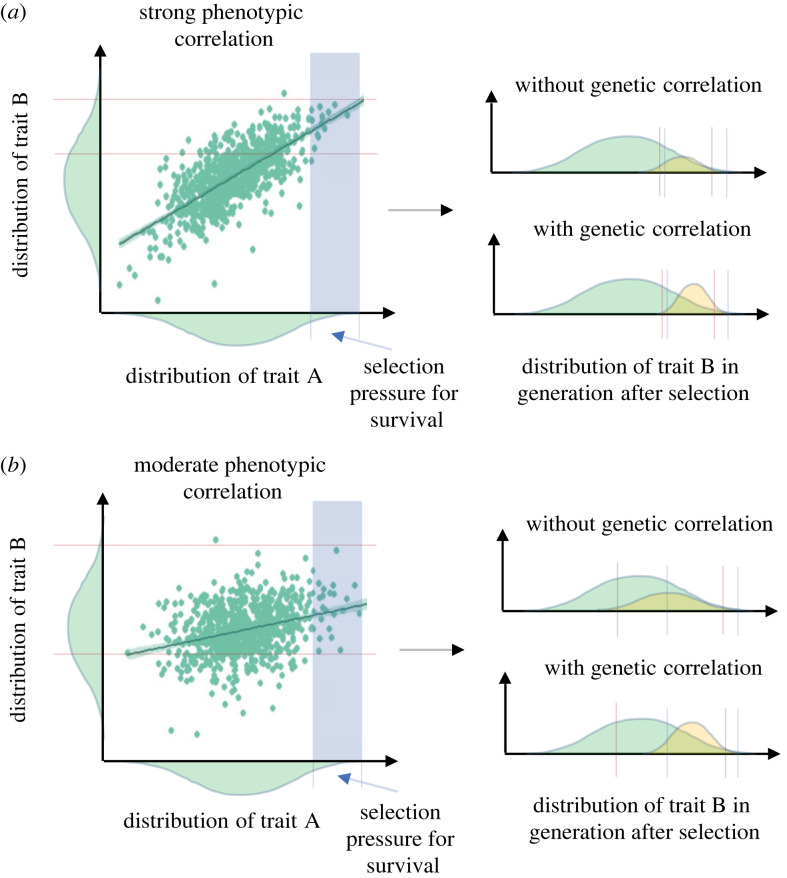
Potential for indirect selection according to the strength of the phenotypic correlation. For illustrative purposes, an extreme selection scenario is depicted where there is a threshold for survival and reproduction, as opposed to a selection gradient. Plots were made using the correlation data between standard metabolic rate and total length (for the strong phenotypic correlation) or specific growth rate (for the moderate phenotypic correlation). (*a*) With a strong phenotypic correlation between traits A and B, selection pressure on trait A could lead to some selection pressure on trait B, as only a small proportion of the phenotypic distribution of trait B would also be under selection and not all the individuals from this part of the phenotypic distribution would be selected, even without genetic correlation. With significant genetic correlation between the traits, trait B would also be selected for by the selection on trait A. (*b*) With moderate correlation between traits A and B, selection pressure on trait A would be unlikely to lead to selection on trait B, as a large proportion of the phenotypic distribution of trait B would be selected and many of the individuals from this part of the phenotypic distribution would not be selected without genetic correlation. With significant genetic correlation between the traits, trait B would be more strongly selected for by the selection on trait A. The darker straight line represents the linear regression between traits A and B. The blue shaded areas correspond to the extreme case of selection pressure for trait A that allow for the survival of only a small proportion of the population. The dashed lines delimitate the part of the phenotypic distribution under potential selection for trait A (blue dashed lines) and trait B (red dashed lines) for the same individuals. The green distribution represents the current phenotypic distribution of the trait, while the yellow distribution represents the expected distribution after selection. (Online version in colour.)

Phenotypic correlations that are not supported by genetic correlation might suggest that the phenotypic correlation is reflective of environmental effects and dependent on the environment in which individuals are measured. The lack of genetic correlations could thus partially explain why, despite the observed phenotypic correlations between metabolic rates and physiological or behavioural traits, as predicted by POLS, an increasing number of studies are questioning the general prevalence of these phenotypic correlations [5,10,11] that could be mainly modulated by environmental factors. In such cases, selection on a trait that is only environmentally linked to another trait may not induce indirect selection on the genetic basis of the other trait, nor constrain its evolution. This is especially the case when negative or non-existent genetic correlations between traits are masked by strong environmental effects [15,30,53], as we see in our data. Abundant food resources could lead to a high metabolic rate being associated with risk-taking, to fuel individual metabolism, for instance. However, selection in the form of higher predation with high risk-taking would not induce strong selection on the genetic basis of metabolic rate, thus leading to limited potential for correlated evolution. The outcome would also depend on the strength of the phenotypic correlation, as selection on traits with very strong, perhaps functionally based, phenotypic correlations could probably lead to direct selection on both traits and, therefore, their evolution if the traits possess genetic variance, while the evolutionary outcome of weak or moderate phenotypic correlation would be more unlikely (figure 3).

## 4. Accuracy of predicting genetic correlations from phenotypic correlations

**Figure 4.**
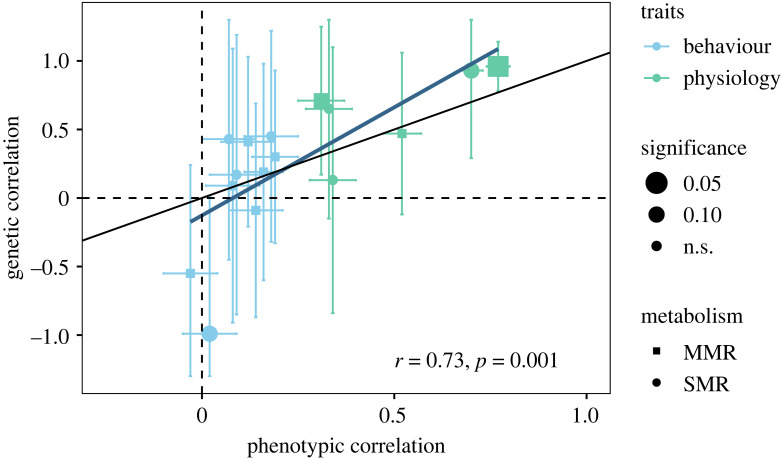
Correlation between phenotypic and genetic correlation between standard metabolic rate (SMR, circle) or maximum metabolic rate (MMR, square) with physiology (green) or behaviour (blue) traits. The significance of the genetic correlation is represented by the size of the symbol. Both 95% confidence intervals for phenotypic and genetic correlations are represented, confidence intervals overlapping the dashed line reflect estimates not different from 0. The black straight line represents the 1 : 1 correlation slope. For illustration purpose, the blue straight line represents the line of best fit between the phenotypic and genetic correlation (generalized linear model: *n* = 16, *F*_1,14_ = 16.31, *p* = 0.001). (Online version in colour.)

To further examine whether the phenotypic correlations could still be used to predict genetic correlations in our case study, we estimated the relationship between the phenotypic and genetic correlations. The phenotypic gambit was only partially supported and failed at estimating genetic correlation. Phenotypic and genetic correlations were strongly and positively correlated using Pearson correlation (figure 4), with a coefficient (*r*) of 0.73, which is in the same order of magnitude as previous studies [23,29]. In addition, the direction of the correlations was mainly matching between the phenotypic and genetic correlations. Overall, therefore, the measurement of phenotypic correlations may give some limited information on the direction of genetic correlations and evolutionary potential, as also previously suggested [23].

However, such extrapolation would need to be used with caution. Because the slope of the regression (1.6, higher than previously reported in other studies [23,29] and different from 1 : 1) suggest that the correlation between the phenotypic and genetic correlations among the metabolic, physiological and behavioural traits fails as a precise estimator of the genetic correlation between the traits [29]. The variation around the slope was also so high that it would be difficult to predict the magnitude of the genetic correlations based on the regression. In addition, the regression might not be linear, revealing that predictions would be highly sensitive to the precision of the predictor variable. Overall, only strong phenotypic correlations (mainly between the metabolic rates and length) were supported by significant genetic correlations. The majority of the phenotypic correlations that were weak or medium (mainly between the metabolic rates and behavioural traits) were not supported by genetic correlations or tended to be supported by negative genetic correlation (especially between the metabolic rates and the distance from centre). While the phenotypic gambit is partially supported, for the direction of the correlation, a precise estimate of the significance and magnitude of the genetic correlation is limited, as also previously reported [23,27,53]. Therefore, more than the significance of the phenotypic correlation among the traits, the strength and confidence intervals of the correlation needs to be taken into consideration to predict potential genetic correlation and indirect selection response among traits when genetic correlations are impossible to estimate.

## 5. Conclusion

Overall, we show that metabolic, physiological and behavioural traits can evolve in response to selection as they possess significant additive genetic variance for selection to act upon. Our results also suggest that selection and evolution of metabolic traits could induce indirect selection mainly on growth-related traits, but not on swimming performance, risk-taking or sociability, even if their phenotypes covary. For those traits, evolution would be more likely to be the result of direct selection on both phenotypes. We also highlight that the phenotypic correlations among those traits can be informative of genetic correlations and evolutionary trajectories to only a limited degree, specifically the direction of the correlation, but are misleading with regard to the magnitude of the genetic correlation.

Further studies are needed to better understand the evolutionary outcomes of variation in metabolic rates within species. Although estimating phenotypic correlations can be useful, proper estimation of the genetic correlation is needed to predict evolutionary outcomes on multivariate phenotypes and determine whether traits possess sufficient shared additive variance over which selection could act to allow potential indirect response to selection and evolution. These considerations are especially important in the study of metabolic rates, which are the culmination of whole-animal energy expenditure across multiple traits and levels of organization, from cellular processes to behaviour. Specific considerations for links between metabolic rates and other traits include the following:

(i) metabolic rates, and physiological or behavioural traits, can be highly dependent on the environment, owing to their high plasticity in response to numerous factors including temperature, feeding-history, training effects and external stressors. Depending on the environment in which traits have been measured, the strength and direction of the phenotypic covariation between traits can change [6,11–13];
(ii) similarly, genetic correlations can also be context-dependent, through gene-by-environment interactions [32]. Therefore, the degree of selection on traits can vary in response to the environmental conditions, and environmental covariation may mask genetic correlations and potential constraints on the evolution of traits. It is thus important to study relationships among metabolism and physiology or behaviour across a range of environmental conditions to better assess what suites of traits are more advantageous under specific conditions and which conditions can drive or limit their correlated evolution;
(iii) links between metabolic rates and growth have been documented in several studies, and the result here suggests a genetic basis to this relationship given the observed links between MMR and growth and SMR and length. Nonetheless, the effects of the environment on plasticity of growth rates (e.g. owing to food availability or thermal variation), may still lead to variable phenotypic correlations between metabolic rates and growth but also variable degrees of indirect selection or evolutionary change depending on the gene-by-environment interactions; and
(iv) if not accounted for, the effects of body mass on metabolic rates can inflate phenotypic correlations and repeatability (sometimes used as proxy for heritability), potentially causing an overestimation of indirect selection effects. However, it may also be argued that given the strong phenotypic correlation between body mass and metabolic rate, and the genetic correlations underlying metabolic rates and growth and size, selection may act directly on the integrated phenotype of mass and metabolic rate together. Researchers must carefully consider their research question and environmental context when examining the combined and singular evolutionary effects of body mass and metabolic rates on a case-by-case basis.

Finally, it should also be noted that each population and species may evolve their own genetic structure linking metabolism to other fitness-related traits, limiting the extrapolation from one population to another one. We encourage additional studies that follow the example we present here, to further understand species and population-level diversity in phenotypic and genetic correlations, to further our knowledge of the mechanisms of indirect selection in the context of environmental change.

## Ethic

The experiment was carried out under license 60/4461 from the Home Office.

## Data Availability

Data that support the findings of this study are openly available from the Enlighten digital repository: https://doi.org/10.5525/gla.researchdata.1167 [32]. Supplementary material is available online [54].
